# Tilting Plant Metabolism for Improved Metabolite Biosynthesis and Enhanced Human Benefit

**DOI:** 10.3390/molecules200712698

**Published:** 2015-07-13

**Authors:** Bhekumthetho Ncube, Johannes Van Staden

**Affiliations:** Research Centre for Plant Growth and Development, University of KwaZulu-Natal Pietermaritzburg, Private Bag X01, Scottsville 3209, South Africa; E-Mail: ncubeb@ukzn.ac.za

**Keywords:** bioactivity, biosynthesis, metabolism, plant secondary metabolite

## Abstract

The immense chemical diversity of plant-derived secondary metabolites coupled with their vast array of biological functions has seen this group of compounds attract considerable research interest across a range of research disciplines. Medicinal and aromatic plants, in particular, have been exploited for this biogenic pool of phytochemicals for products such as pharmaceuticals, fragrances, dyes, and insecticides, among others. With consumers showing increasing interests in these products, innovative biotechnological techniques are being developed and employed to alter plant secondary metabolism in efforts to improve on the quality and quantity of specific metabolites of interest. This review provides an overview of the biosynthesis for phytochemical compounds with medicinal and other related properties and their associated biological activities. It also provides an insight into how their biosynthesis/biosynthetic pathways have been modified/altered to enhance production.

## 1. Introduction

Being sessile organisms, plants constantly interact with a multitude of variable and potentially damaging factors in their habitats that range from an abiotic to biotic nature. The survival of floral diversity within ecosystems thus requires elaborate mechanisms of defence. Among these, chemical defences represent the main trait of an innate immune system to cope with the hostile environment. Their metabolic plasticity evolves and exploits a range of inherent systems to create a rich repertoire of complex metabolites that hold adaptive significance for survival in diverse ecological niches.

These phytochemical derivatives of secondary metabolism confer a multitude of adaptive and evolutionary advantages to the producing plants [1]. As a strategy for survival and for the generation of diversity at the organismic level, the ability to synthesise particular classes of secondary metabolites is often restricted to selected taxonomic groups and often imparts a species-specific chemical “signature” for a specific habitat [1,2]. The two recognisable and most characteristic features of secondary metabolism as can be deduced from the descriptions herein are: structural diversity and high intraspecific variability, attributes of which can be described as its inherent lineaments. Apart from regulating the interaction between plants and their environment (biotic and abiotic), plant secondary metabolites also mediate certain physiological aspects of plant growth and development, symbiosis, and reproduction, and are important structural components of the secondary cell wall (lignin) [3,4]. It is against this background that Hartmann [5] describes secondary metabolites on the basis of functionality. The author regards secondary metabolism as the functional level of plant metabolism that is dispensable for growth and development but indispensable for the survival of the species. 

A significant number of these biologically active phytochemicals have historically been exploited for a number of purposes and most prominently in the search for pharmaceutical agents, an aspect that gives credence because of their diverse chemical attributes. Their production is tightly regulated at the level of expression of the biosynthetic genes by developmental and tissue-specific factors as dictated by external signals [6]. The high degree of plasticity of secondary metabolism which, in contrast to primary metabolism, allows for structural and chemical modifications with almost unlimited restrictions is emphasised as a mechanistic basis for the generation of chemical diversity. The diverse molecular changes that are associated with the metabolism are understood to be preserved genetically, functionally and structurally to confer selective and adaptive advantage on the biosynthetic hosts in diverse ecosystems [1,7]. Benderoth *et al*. [8] pointed a combination of gene duplication, neofunctionalisation, and positive selection as mechanisms for the evolution of this diversity. Research evidence posits the basis of this genetic variation as being responsible for generating terrestrial organic diversity in response to plant-environment interactions. It is thus upon this principle that classic hypotheses that seek to explain this vast metabolic diversity propose a stepwise and reciprocal process of adaptation and counter-adaptation between plants and their natural enemies as shaped by mutual selection are rooted [8,9,10].

Despite this immense structural diversity, secondary metabolites derive their synthesis from limited products of primary metabolism. The ongoing research efforts have elucidated the basic biochemistry and molecular biology of some biosynthetic pathways of secondary metabolism, with most of the findings supporting that the diversification of secondary metabolism originates from the elaboration of a few central intermediates [2,7,10,11]. Furthermore, the vestigial structural and mechanistic traits that characterise the biosynthetic enzymatic pathways during the diversification of substrate to product specificities is remarkably conserved via a few but complex biogenetic routes. The catalytic landscape adapts all possible lineages as the enzyme family used simple transformations in order to use new substrates and ensure product selectivity. A better understanding of the enzymatic machinery that underlies the often complex pathways of phytochemical biosynthesis is becoming clearer with advances in molecular technology and genomics.

The heritable and adaptable nature of this metabolism and subsequent chemical variation presents a unique biogenic resource that man continues to rationally engineer using the unique perspectives of evolution, genomics and structural biology to create novel compounds. In this review, we discuss the biosynthesis and biological activity of some selected classes of secondary metabolites. We also provide insight into the approaches used to alter metabolism for human-directed production compound. 

## 2. Secondary Metabolism and Metabolites

**Figure 1:**
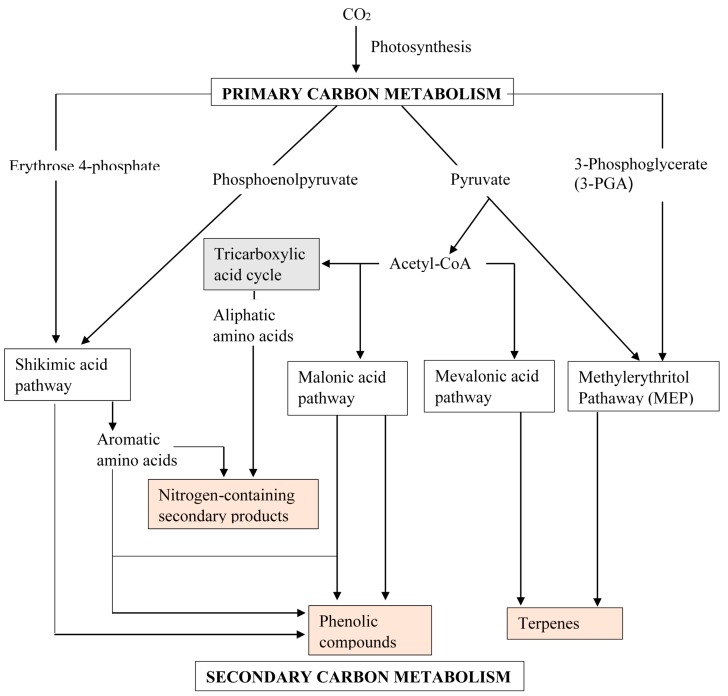
A simplified general overview of the biosynthetic pathways involved in the biosynthesis of secondary metabolites showing a tight association with the product of primary/central metabolism. Pink boxes represent secondary metabolites while primary metabolites are without boxes. The pathways in unshaded boxes represent secondary metabolism and the one shaded grey is part of the primary metabolism (most not shown).

The interdisciplinary concerted research efforts and successes in identifying genes and enzymes involved in plant secondary metabolism has brought about our current understanding of metabolic pathways leading to the biosynthesis of secondary metabolites. Secondary metabolism comprises a coordinate series of coupled enzymatic conversions that utilises limited products of primary/central metabolism as substrates/intermediates. Secondary metabolism uses highly organized systematic mechanisms that integrate into developmental, morphological and biochemical regulatory patterns of the entire plant metabolic network. The inevitable link between metabolic fluxes of central metabolism and the biosynthesis of secondary metabolites (Figure 1) further substantiates the existence of coordinated gene expression networks at the interface of the two metabolisms [12,13,14,15]. Accumulating evidence suggests that many transcriptional factors (TFs) coordinate the transcriptional activation of secondary metabolism genes concurrently with the expression of genes in upstream pathways of primary metabolism [12,16,17]. For example, regulation of glucosinolate biosynthesis in *Arabidopsis thaliana* is not restricted to the metabolic space surrounding its biosynthesis but rather tightly linked to more distal metabolic networks of primary metabolism [14]. Transgenic *A. thaliana* overexpress two clades of genes, *ATR1*-like and *MYB28*-like genes that regulate the aliphatic and indole glucosinolate biosynthetic pathways. Transgenic *A. thaliana* concomitantly induced genes are involved in the sulphur assimilation pathways as well as in the formation of precursor molecules for the biosynthesis of both glucosinolates. In this same system, all genes responsible for the enzymatic reaction steps of the TCA cycle from oxaloacetate to methionine were induced concurrently with genes encoding enzymes that regulate the committed steps in aliphatic glucosinolate biosynthesis in plants overexpressing the TFs. These changes were accompanied with changes in the levels of the affected central metabolites. The relatively broad view of transcripts and metabolites altered in transgenic plants overexpressing the different factors (*ATR1* and *MYB28*) [14,17] underlined novel links of glucosinolate metabolism to additional metabolic pathways, including those of jasmonic acid, folate, benzoic acid, and various phenylpropanoids. While there is no evidence that these sets of TFs bind directly to the upstream regions of genes belonging to the central metabolic pathways, the findings pointed to a shared and coordinated transcriptional regulation of primary and secondary pathways [14,17,18,19].

To address the potential breadth of transcriptional regulation that alters accumulation of defensive glucosinolate metabolites in *A. thaliana* across environmental stresses, Li *et al.* [17] further identified hundreds of unique potential regulatory interactions with a nearly complete complement of 21 promoters for the aliphatic glucosinolate pathway. High-throughput phenotypic validation demonstrated that more than 75% of the tested TF mutants significantly altered the accumulations of glucosinolates and that these were conditional upon the environment and tissue type. Results suggest that TFs allow the plant to tune its defences to adjust to the environment. The conclusion drawn is that defence chemistry within the plant has a highly intricate transcriptional regulatory system that may allow for the optimisation of defence metabolite accumulation corresponding to specific environmental cues. Similarly, the phenylpropanoid metabolism, in which phenolic compounds are derived, is regulated by coordinate changes of gene expression accompanied by changes in the expression of genes that encode enzymes in primary metabolism [20,21,22]. Many of the genes encoding the enzymes of phenylpropanoid metabolism contain, within their promoters, well conserved motifs that conform to the motifs recognised by plant MYB TFs and some of which are able to transactivate genes encoding phenylalanine ammonia-lyase (PAL) in primary metabolism [22,23,24]. Henkes *et al.* [20] identified transketolase activity as an important determinant of photosynthetic and phenylpropanoid metabolism and showed that the provision of precursors by primary metabolism co-limits flux into the shikimate pathway and phenylpropanoid metabolism. A slight modification in transketolase activity significantly alters phenylpropanoid metabolism [20]. Similarly, in terpenoid indole alkaloid biosynthesis in *Catharanthus roseus*, overexpression of *ORCA3*, a jasmonate-responsive AP2/ERF-domain family TF, led to an induction of genes encoding two enzymes (anthranilate synthase and D-1-deoxyxylulose 5-phosphate synthase) involved in central metabolism [25,26]. *ORCA3* overexpression resulted in enhanced expression of several metabolite biosynthetic genes and, consequently, in increased accumulation of terpenoid indole alkaloids [25].

At the helm of metabolic engineering for improved secondary metabolite biosynthesis, is the consideration of the entire metabolic network to redirect central metabolites into secondary metabolites without compromising plant fitness. The fact that secondary metabolites are derived from several different precursors and that the intersection of their metabolism with other metabolic pathways produces key metabolic intermediate compounds suggests complex regulation of their production. Such regulatory networks should modulate levels of each metabolite either coordinately or separately to optimise fitness, as dictated by developmental and/or environmental cues [27]. Against this background, it is suffice to theorise that the availability of resources (carbon, nitrogen, and sulphur) that affect specific groups of primary metabolites will consequently impact quantitative and qualitative production of secondary metabolites. With the current level of functional understanding, a more complete appreciation of complex secondary biosynthetic pathways, together with mechanisms of individual biosynthetic reactions, is within reach. The combined information can provide a more practical and rational metabolic engineering basis for the production of pharmaceutically important and novel metabolites from plants.

There is no fixed, commonly agreed upon system for classifying secondary metabolites. However, based on their biosynthetic origins, they can be broadly divided into three main groups: nitrogen or sulphur containing compounds such as alkaloids and glucosinolates phenolic compounds, and terpenoids/isoprenoids, respectively. A number of these phytochemicals have established ecophysiological and defence adaptive roles in plants. A reservoir of more than 200,000 known secondary metabolites, with many more that continue to be discovered, provides mankind with a biogenic resource to exploit for pharmaceuticals. We now describe briefly the biosynthetic pathways of each of the main groups of secondary metabolites and then provide some mechanisms through which these can be manipulated to produce compounds beneficiary to humans.

### 2.1. Alkaloids

Alkaloids encompass an enormous class of approximately 12,000 low-molecular weight natural products [28]. The principal requirement for classification as an alkaloid is the presence of a basic nitrogen atom at any position in the molecule, which does not include nitrogen in an amide or peptide bond [29]. As implied by this exceptionally broad definition, alkaloids form a group of structurally diverse and biogenically unrelated molecules. As opposed to most types of secondary metabolites whose similar chemical structures are derived from related biosynthetic pathways, the many classes of alkaloids have unique biosynthetic origins [30]. Alkaloid biosynthesis and accumulation are associated with a variety of cell types in different plants, including epidermis, endodermis, pericycle, phloem parenchyma, phloem sieve elements and companion cells, specialized mesophyll, and laticifers. A common paradigm is the involvement of multiple cell types and the implied transport of pathway intermediates and/or products [28]. The complex intracellular compartmentation of alkaloid biosynthesis is thought to have occurred as a consequence of adapting compartmented reactions of primary metabolism to participate in alkaloid biosynthesis [31]. The subcellular trafficking of pathway intermediates also creates an important level of metabolic regulation that could not occur if enzymes and substrates diffused freely in the cytosol. Alkaloids are commonly synthesised from amino acids as starting precursor molecules, although some purine-derived alkaloids are also known. The substrate starting material typically defines the structural class of the alkaloid [30]. Owing to their potent biological activity, many of the approximately 12,000 known alkaloids have been exploited as pharmaceuticals, stimulants, narcotics, and poisons.

Although the exact roles of many alkaloids are not well understood, the compounds are believed to play an important ecological role, enabling the producing organism to interact defensively with its environment. They confer a survival benefit through their ability to bind to cellular targets in antagonistic organisms [32,33]. Due to the toxic nature of most alkaloids, their biosynthesis provides a general defensive mechanism for the producing organism. One such example is caffeine, which has been demonstrated to act as a natural insecticide in plants. When the three *N*-methyltransferase genes involved in caffeine biosynthesis were overexpressed in tobacco, the resulting increase in caffeine production improved the tolerance of the plants to certain pests [34,35]. Despite the metabolic diversity and chemical complexities in alkaloid biosynthesis, a number of technical breakthroughs have only recently contributed to significant advancements in understanding alkaloids. This has largely been made possible by our ability to investigate secondary metabolism from a combined biochemical, molecular, cellular, and physiological perspective. Table 1 represents some of the biologically active plant-derived alkaloids utilised in modern medicine.

**Table 1:** Biologically active alkaloids exploited in modern medicine.

Alkaloid	Plant Source	Pharmaceutical Use
Tropane and Nicotine		
Atropine	*Hyoscyamus niger*	Anticholinergic, antidote to nerve gas poisoning [36]
Cocaine	*Erythroxylon coca*	Topical anaesthetic, potent central nervous system stimulant, and adrenergic blocking agent; drug of abuse [30,37]
Codeine	*Papaver somniferum*	A nonaddictive analgesic and antitussive [30]
Morphine	*Papaver somniferum*	Powerful narcotic analgesic, addictive drug of abuse [30]
Nicotine	*Nicotiana tabacum*	Highly toxic, causes respiratory paralysis, horticultural insecticide; drug of abuse [37]
Scopolamine	*Hyoscyamu. niger*	Powerful narcotic, used as a sedative for motion sickness [36]
(+)-Tubocurarine	*Chondrodendron tomentosm*	Nondepolarising muscle relaxant producing paralysis, used as an adjuvant to anaesthesia [37]
**Amarryllidaceae**		
Galanthamine	*Galanthus woronowii*	Used in the treatment of Alzheimer’s disease [37]
**Piperidine**		
Coniine	*Conium maculatum*	An extremely toxic alkaloid, causes paralysis of motor nerve endings, used in homeopathy in minute doses [38]
**Terpenoid Indole Alkaloids**		
Ajmaline	*Rauwolfia serpentina*	Antiarrythmic that functions by inhibiting glucose uptake by heart tissue mitochondria [37]
Camptothecin	*Camptotheca acuminata*	Potent anticancer agent [39,40]
Quinine	*Cinchona officinalis*	Traditional antimalarial, important in treating *Plasmodium falciparum* strains that are resistant to other antimalarials [41]
Strychnine	*Strychnos nuxvomica*	Tetanic poison, rat poison, used in homeopathy [37]
Vinblastine	*Catharanthus roseus*	Antineoplastic used to treat Hodgkin’s disease and other lymphomas
Vincristine	*Catharanthus roseus*	Am antitumor and chemotherapeutic agent [37]
**Purine**		
Caffeine	*Coffea arabica*	Used as a central nervous system stimulant [42,43,44]
**Pilocarpus**		
Pilocarpine	*Pilocarpus jaborandi*	Peripheral stimulant of the parasympathetic system, used to treat glaucoma [37]
**Ipecac**		
Emetine	*Uragoga ipecacuanha*	Orally active emetic, amoebicide [37]
**Benzophenanthridine**		
Sanguinarine	*Eschscholzia californica*	Antibacterial showing antiplaque activity, used in toothpastes and oral rinses [37]

#### 2.1.1. Tropane and Nicotine Alkaloids

The tropane class of alkaloids are an important class of plant-derived anticholinergic compounds, such as hyoscyamine and scopolamine and the narcotic tropical anesthetic cocaine, that occur mainly in Solanaceae. The central nervous system stimulant cocaine is a tropane which is found outside Solanaceae (in *Erythmxylon coca*). Ornithine and/or arginine serve as the first precursor molecules from which putrescine is derived through decarboxylation. The early steps of tropane alkaloid and nicotine biosynthesis are also common to polyamine metabolism. The first committed step for the two alkaloid pathways begins with the methylation of putrescine to *N*-methylputrescine by putrescine *N*-methyltransferase which is subsequently deaminated by a diamine oxidase to 4-aminobutanol. The 4-aminobutanol then undergoes spontaneous cyclisation to form the reactive *N*-methyl-Δ^1^-pyrrolinium cation, a central intermediate for the biosynthesis of tropane alkaloids and nicotine [45]. For nicotine, the cation is condensed with nicotinic acid to form 3,6-dihydronicotine, which subsequently undergoes dehydrogenation to nicotine. In the case of tropane alkaloids, the cation is thought to condense with acetoacetic acid to yield hygrine as a precursor of the tropane ring [28]. Tropinone is located at a branch point of this pathway and is the first intermediate with a tropane ring [28,36]. The enzymatic activity of these reactions have, however, not been demonstrated. Two related dehydrogenases, tropinone reductase I (TR-I) and tropinone reductase II (TR-II), reduce the 3-keto group of tropinone to the 3α- and 3β- groups of the stereospecific alkamines tropine and ψ-tropine, respectively [36]. The exchange of various domains of TR-I and TR-II creates a series of chimeric enzymes at the C terminus and it was demonstrated that 120-amino acid residue peptide of each reductase determines the stereospecificity of the reaction it catalyses [46]. Hyoscyamine is then produced by the condensation of tropine and the phenylalanine-derived intermediate tropic acid and can be converted to its epoxide scopolamine.

**Figure 2:**
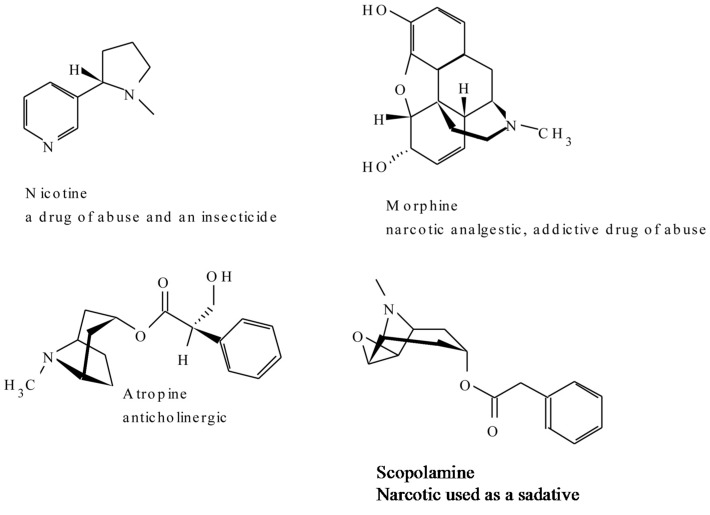
A few representative structures of the tropane and nicotine alkaloids.

One of the examples of the biologically active members of this class of alkaloids is scopolamine which is commonly used today in the form of a transdermal patch to combat motion sickness. Cocaine, which served as a “lead,” a starting structure that medicinal chemists modified for the development of an optimised drug, synthetic topical anesthetics, is also one other example that comes into mind. *Datura* leaves are usually smoked for the hallucinogenic effects of scopolamine [36]. Cocaine is illicitly applied to mucus membranes for its addictive stimulatory effects. Figure 2 shows some of the representatives of the compounds in this group (Figure 2).

#### 2.1.2. Amaryllidaceae Alkaloids 

**Figure 3:**
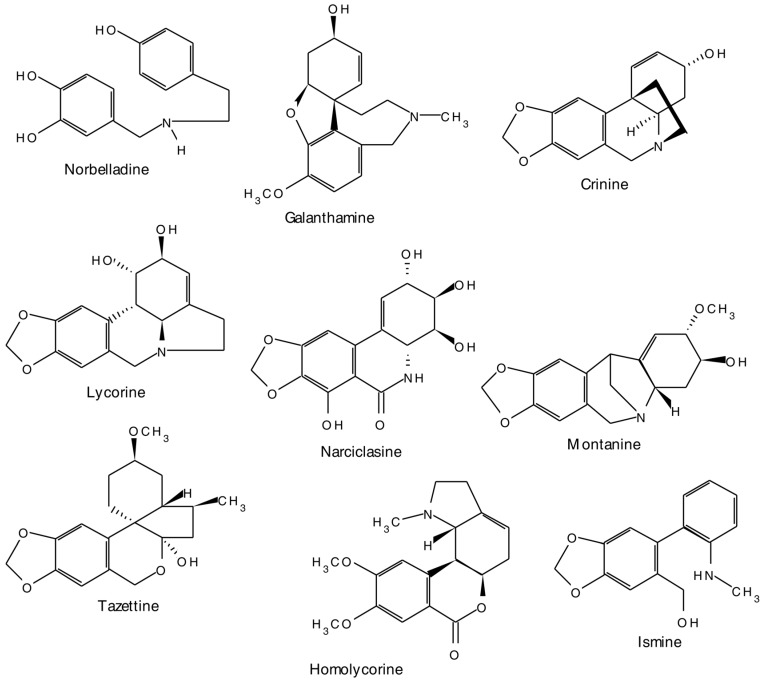
A few representative structures of the Amaryllidaceae group of alkaloids.

The Amaryllidaceae alkaloids, represent a group of isoquinoline alkaloids, which are produced almost exclusively by members of the Amaryllidaceae family. Although there are several other alkaloids having structures derived from these main molecular frameworks, Amaryllidaceae alkaloids may be classified into nine skeletally homogenous subgroups. Representative alkaloids from each of these classes include norbelladine, lycorine, homolycorine, crinine, ismine, tazettine, narciclasine, montanine, and galanthamine [37] (Figure 3).

Although the precise chemistry and the nature of enzymes involved in the early steps of isoquinoline alkaloid biosynthesis are less well characterized, the compounds are known to be formed biogenetically by intramolecular oxidative coupling of norbelladine and its derivatives [47,48]. The biosynthetic process is initiated by the formation of norbelladine [49], which later undergoes oxidative phenolic coupling, and subsequent transformation into the final alkaloid structures [31]. Phenylalanine and tyrosine are the precursors of norbelladine. Key regulatory functions are often associated with enzymes, such as PAL that operate at the interface of primary and secondary metabolism [50]. Decarboxylation of these amino acids is an important step in the biosynthetic pathway of this group of alkaloids. Oxidative phenol couplings, which give rise to new skeletons, is responsible for the wide structural diversity in the isoquinoline group of alkaloids.

The pharmacological uses of many of this group of alkaloids are well defined. These include, the potent inhibition of acetylcholinesterase (with potential application to Alzheimer’s disease therapies), cytotoxicity, antibacterial, antiviral, anti-inflammatory, antiparasitic, antihistaminic, antiproliferative, anticancer and adrenergic activity, antineoplastic against murine P-388 lymphocytic leukemia, and treatment of mental disorders and age-related dementia [51,52,53,54]. The pharmaceutical potential of the Amaryllidaceae alkaloids has been recognised through the commercialisation of galanthamine as an Alzheimer’s drug due to its potent and selective inhibitory activity against the enzyme acetylcholinesterase.

The lycorane derivatives pancratistatin and narciclasine, hold promising chemotherapeutic potential due to their potent, selective anticancer properties, although the specificity of the compound to cancer cells and its mechanism of action remain unknown [55,56]. In these studies, pancratistatin had a greater specificity than etoposide (VP-16) or paclitaxel in selectively inducing apoptosis. Narciclasine disrupts organisation of the actin skeleton in cancer cells at very low concentrations (30–90 nM) [57,58] as well as increasing survival in preclinical models of human glioblastoma multiforme by markedly decreasing mitotic rates without inducing apoptosis [59]. Narciclasine is available in relatively large quantities from many of the Amaryllidaceae, including common varieties of *Narcissus pseudonarcissus.* With galanthamine and pancratistatin established as leads, and owing to the wide chemical diversity and the limitless ability of the Amaryllidaceae members to produce these chemical compounds, the Amaryllidaceae alkaloids thus provide a rich and accessible platform for the discovery of novel and innovative drugs. 

#### 2.1.3. Terpenoid Indole Alkaloids

These alkaloids are distributed mainly in the Apocynaceae, Rubiaceae, Loganiaceae, and Nyssaceae families [60]. Examples of alkaloids in this class include quinine, ajmalicine, strychnine, and vincamine. Terpenoid indole alkaloids consist of an indole moiety provided by tryptamine and a terpenoid component derived from the iridoid glucoside secologanin. Tryptamine is derived from decarboxylation of tryptophan by tryptophan decarboxylase, which plays a key role in terpenoid indole alkaloid biosynthesis by linking primary and secondary metabolism. López-Meyer *et al*. [41] identified the differential expression of the enzyme tryptophan decarboxylase in *Camptotheca acuminata* during stress and development. These results suggest that the enzyme plays a dual role in primary and secondary defence function.

The first committed step in secologanin biosynthesis is the hydroxylation of geraniol to 10-hydroxygeraniol, with the conversion of loganin to secologanin representing the last step in the pathway [60]. Secologanin is derived from the triose phosphate/pyruvate pathway and its production is thought to play a regulatory role in terpenoid indole alkaloid biosynthesis [61]. Secologanin is a biogenetic key isoprenoid glucoside, a polyfunctional molecule which occupies a central position in several biosynthetic pathways and acts as a starting compound for a multitude of other natural products [61]. The molecule is the ultimate precursor of the C9-C10 moiety common to the majority of the indole alkaloids and of some quinoline and isoquinoline alkaloids [61]. Strictosidine, the common precursor to all terpenoid indole alkaloids, is formed by a Pictet-Spengler condensation of tryptamine and secologanin catalised by strictosidine synthase. The subsequent removal of the strictosidine glucose moiety leads to a strictosidine-derived aglycone which is then converted via several unstable intermediates to dehydrogeissoschizine and represents a key branch point intermediate that leads to several diverse terpenoid indole alkaloid pathways [28].

**Figure 4:**
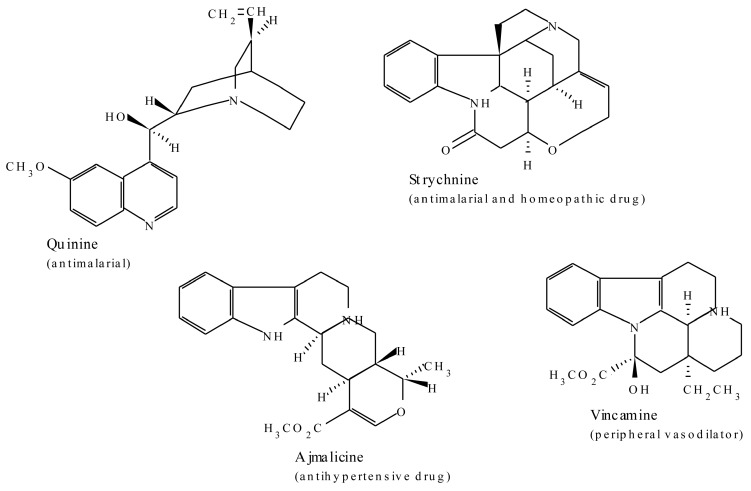
Examples of plant-derived bioactive terpenoid indole alkaloids.

Many of the terpenoid indole alkaloids are physiologically active in mammals. Camptothecin, isolated from *Camptotheca acuminate* (Nyssaceae), possesses anti-tumour activity which is due to its ability to inhibit DNA topoisomerase [39]. Through inhibition of Tat-mediated transcription [40], the compound also inhibits anti-retroviruses such as HIV and the equine infectious anaemia virus [39,62] and has also shown good activity against parasitic trypanosomes and *Leishmania* [63]. The three antitumor and chemotherapeutic agents, vincristine, vinblastine, and ajmalicine isolated from *Catharanthus roseus,* are examples of the terpenoid indole group of alkaloids. Quinine from *C. officinalis* is an antimalarial drug and strychnine is a rat poison and homeopathic drug from *Strychnos nuxvomica.* Topotecan and irinotecan are to date some of the US Food and Drug Administration (FDA) approved agents for the treatment of ovarian and colon cancer [41]. Some examples of bioactive members of this group of alkaloids are represented in Figure 4.

#### 2.1.4. Purine Alkaloids

**Figure 5:**
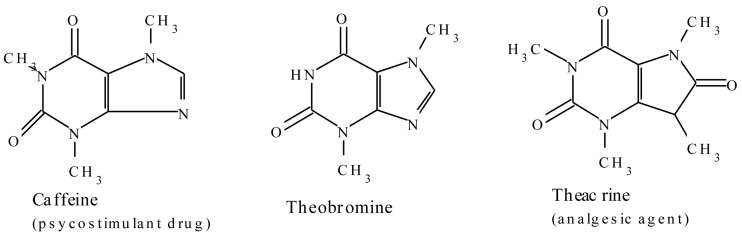
Examples of the members of the plant-derived purine group of alkaloids.

Purine alkaloids are widely distributed in the plant kingdom and examples include caffeine, theobromine, and theacrine (Figure 5). The alkaloids are derived from purine nucleotides. In caffeine, xanthosine forms the starting point for the major biosynthetic route and proceeds through three *N*-methylations via 7-methylxanthosine, 7-methylxanthine, and theobromine [64,65] with a number of other minor pathways identified [65,66,67]. Caffeine is known to have sensory and stimulatory effects when consumed as a psychostimulant drug in coffee (*Coffea arabica* and *Coffea canephora*), tea (*Camellia sinensis*), chocolate, and soft drinks. The mechanism of bioactions are thought to be mediated via a blockade of the adenosine A_1_ and A_2A_ receptors. Adenosine is a modulator of CNS neurotransmission and its modulation of dopamine transmission through A_2A_ receptors is implicated as the mechanism of psychomotic effects of caffeine [42,43,44]. The alkaloid aids in concentration through alleviating fatigue and increasing wakefulness [68]. Xanthines also produce numerous physiological effects that include positive inotropic and chronotropic effects on the heart, decreased airway resistance in the lung, and respiratory stimulation [44,69].

### 2.2. Phenolic Compounds

**Figure 6:**
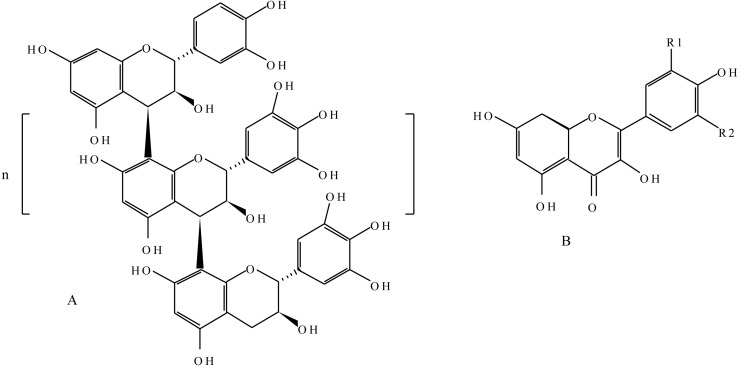
(**A**) Chemical structure of a proanthocyanidin consisting of the catechin and epicatechin polymeric units, where “n” is any number that makes up the polymer; (**B**) Generic structure of a flavonoid. Kaempferol, R1 = H, R2 = H; quercetin, R1 = OH, R2 = H; myricetin, R1 = OH, R2 = OH.

The successful evolutionary adaptation of plants to land can largely be attributed to the massive formation of “plant phenolic” compounds. Although most of these substances assumed cell wall structural roles, a number of adaptive, defensive, and certain distinguishing features exists in some plant species. Phenolic compounds or polyphenols are characterised by at least one aromatic ring bearing one or more hydroxyl substituents, and other functional derivatives such as esters, methyl esters, and glycosides [70]. They are a diverse group of higher secondary metabolites, with derivatives of the pentose phosphate, shikimate, acetate, and phenylpropanoid metabolism, and phenylalanine as the precursor molecule [71,72]. The phenylpropanoid pathway is one of the most important metabolic pathways in plants in terms of carbon flux, with more than 20% of the cell total metabolism going through this pathway [73]. From a polyphenol point of view, the pathway yields, among others, flavonoids, lignans, lignins, and anthocyanins. Key to the biosynthesis is the enzyme PAL, which converts phenylalanine into trans-cinnamic acid by a non-oxidative deamination process. The enzyme plays an important role in controlling the flux into the pathways. The basic building unit of polyphenol is phenol. Although polyphenols are ubiquitous in the plant kingdom, the type of compound produced varies considerably between genera and species [74]. Phenolic compounds form an integral part of the cell wall structure in plants, mainly in the form of polymeric materials such as lignins, which serve as mechanical support and barriers against microbial invasion. They account for about 40% of organic carbon circulating in the biosphere. The compounds exhibit a considerable free radical scavenging activity, determined largely by their reactivity as hydrogen- or electron- donating agents and the stability of the resulting antioxidant-derived radical prevents the oxidation of various food ingredients, particularly fatty acids and oils [70,75]. Flavonoids and tannins are amongst the broad groups of phenolic compounds (Figure 6).

#### 2.2.1. Flavonoids

Built upon a flavone skeleton, flavonoids are the most widespread and diverse class of low molecular weight phenolic compounds [76]. Biosynthetically, they are derived from a combination of the shikimic acid and the acetate pathways. Flavonoids can occur as monomers, dimers, and higher oligomers. They are constituents of a variety of plant parts, including, leaves, fruits, seeds, flowers, and roots, with over 4000 different variants identified [74,76]. Small differences in basic substitution patterns give rise to several subgroups, with the main subclasses being flavonols, flavones, flava-3-ols, isoflavones, chalcones, flavanones, catechins, leucoanthocyanidins, and anthocyanidins [76]. The first committed step of the flavonoid pathway is catalysed by chalcone synthase leading to the condensation of three molecules of acetate-derived malonyl-CoA and one molecule of *p*-coumaryl-CoA to generate a tetrahydroxychalcone. In certain plant species, the coordinated action of this enzyme and an NADPH-dependent reductase generates isoliquiritigenin. Both chalcones can then be converted into aurones, a subclass of flavonoids found in certain plant species. Beyond this point, the next shared step by most of the flavonoid biosynthesis pathways is facilitated by chalcone isomerase, which catalyses a stereospecific ring closure isomerisation step to form naringenin and, less commonly, liquiritigenin. The two flavanones represent the most important branching point in flavonoid metabolism. The generic flavone structure is a illustrated in Figure 6.

Structural and chemical diversity of flavonoids is related to their diverse properties and roles in plants. In the producing plants, flavonoids provide protection against ultraviolet radiation, invading pathogens, and herbivores [77]. One of the ascertained functions of flavonoids in plants is their protective role against microbial invasion [77,78]. The ability of flavonoids to inhibit spore germination of pathogens has been explored and exploited in both traditional and modern human medicine for the treatment of human pathogenic diseases. Numerous flavonoids have been characterised as antifungal, antibacterial, antiviral, anti-inflammatory, antioxidant, antitumor, anti-hepatotoxic, anti-lipolytic, vasodilator, immunostimulant, and antiallergic agents [79,80,81,82,83]. Several consistent lines of evidence support the role of flavonoids in radical scavenging, chelating, and oxidant activities against various reactive oxygen species (ROS) in animal cells. Consistent with most polyphenolic antioxidants, both the configuration and total number of hydroxyl groups, in addition to the flavan backbone, substantially influence several mechanisms of antioxidant activity. The free radical scavenging capacity of flavonoids can primarily be attributed to this characteristic [76,83].

#### 2.2.2. Tannins

Tannins are phenolic compounds that exhibit complex and highly variable chemical structures. They are broadly categorised into hydrolysable and condensed tannins, based on whether acids or enzymes can hydrolyse the components or whether they condense the components to polymers [84]. Both classes of tannins are rich in highly reactive hydroxyl groups which emanate from each of the benzene ring constituents. These form complexes with proteins, including enzymes [85] and polymers such as cellulose and hemicellulose [86].

Hydrolysable tannins are based upon the fundamental structural unit of gallic acid (3,4,5-trihydroxy benzoic acid) and are almost invariably found as multiple esters with d-glucose to form gallotannins. Derivatives of hexa-hydroxydiphenic acid (ellagitannins) are derived from oxidative coupling of adjacent galloyl ester groups in a polygalloyl d-glucose ester [86,87]. The central compound, pentagalloylglucose, is the starting point for many tannin structures. The compound consists of polyols, such as glucose, surrounded by several gallic acid units. 

Condensed tannins, commonly referred to as proanthocyanidins, are oligomers of 3-flavanols (catechins) and 3,4-flavan-diols (leucoanthocyanidins) linked together by single interflavan carbon to carbon bonds [88]. The flavan-3-ol units are linked principally through the 4 and the 8 positions. The term proanthocyanidin is derived from the acid-catalysed oxidation reaction that produces red anthocyanidins upon heating in an acidic alcohol solution, a reaction that forms the basis of the butanol-HCl assay for proanthocyanidins. Their structures depend upon the nature (stereochemistry and hydroxylation pattern) of the flavan-3-ol starter and extension units, the position and stereochemistry of the linkage to the ‘‘lower’’ unit, the degree of polymerisation, and the presence or absence of modifications such as esterification of the 3-hydroxyl group (Figure 6). Most proanthocyanidins are built from the flavan-3-ols and catechin and epicatechin skeletons [89]. They are the most widespread polyphenols in plants after lignins. In plants, condensed tannins may act as feeding deterrents in reproductive tissues and developing fruit and also impart astringency to fresh fruit, fruit juices, and wine [86]. Tannins are characteristic of the chemical defence of plants and act as quantitative-dosage dependent-barriers to predators that may feed on them [90]. The relevant physiological effects of tannins upon predation is assumed to be derived from their ability to complex with proteinaceous materials.

Due to their antibiotic, antifeedant, or biostatic effects on a variety of organisms that consume them, the chemical properties of both condensed and hydrolysable tannins have been exploited in the discovery of versatile medicinal agents. Tannins are, thus, reported to possess numerous pharmacological properties such as antibacterial, antifungal, antiviral, anti-diarrhoeal, free radical scavenging, immunomodulatory, anti-inflammatory, anti-tumour, and antidote activities [86,91,92,93]. Some, and certainly most, of the beneficial effects which tannins exert as constituents of drugs and herbal remedies may well follow from their interaction with enzymes (proteins) within cell systems [91,94]. Vanden Berghe *et al.* [95] evaluated the claims for antiviral activity made for natural products, including various tannins, derived from over 900 plant species, and concluded that tannins act principally by binding to the virus and/or protein of the host cell membrane and thus arresting adsorption of the virus. Similarly, bacterial and fungal enzymes and toxic proteins may be bound by tannins and inactivated in a similar manner [94]. This propensity to bind to proteins also presumably accounts for the fact that polyphenols inhibit virtually every enzyme that is tested *in vitro* [86,96].

Polyphenol and protein complexation is essentially a surface phenomenon, maximised at or near the isoelectric point of the protein [91]. Interactions are dynamic and time dependent; conformational flexibility in both the polyphenol and the protein are important complimentary factors that lead to strong interactions. Through their aromatic nuclei and phenolic groups, polyphenols act as multidentate ligands on the protein surface and the efficacy of binding increases as the number of polyphenol galloyl groups increases [91].

### 2.3. Terpenes

Terpenes, one of the largest and perhaps most structurally diverse groups of secondary metabolites, are all synthesised from two precursors, dimethylallyl pyrophosphate (DMAPP) and isopentenyl pyrophosphate (IPP). Two independent pathways contribute to the formation of isopenteny1 diphosphate and dimethylallyl diphosphate, the two central building blocks of isoprenoids in higher plants. Plants invariably utilise the mevalonate pathway in the cytosolic compartment and the non-mevalonate pathway in plastids, an aspect that signifies subcellular compartmentalisation of the pathways. In general, the cytosolic mevalonate pathway provides the precursors for sesquiterpenes and sterols, whereas the plastidial methylerythritol pathway (MEP) furnishes the monoterpene, diterpene, and carotenoids [97,98]. Recent evidence suggests the presence of an unidirectional proton symport system in plastid membranes for the export of specific isoprenoid intermediates involved in the metabolic cross talk between cytosolic and plastidial pathways [99,100]. The lack of correlation between gene expression patterns and the accumulation of isoprenoid metabolites indicates that posttranscriptional processes may play an important role in regulating flux through isoprenoid metabolic pathways [100]. Chemical diversity of these compounds emanate from the general isoprenoid pathway by activities of large gene families for two classes of enzymes, the terpene synthases and the cytochrome P450-dependent monooxygenases of the CYP720B group [101]. Subsequent modifications of the basic parent skeletons produced by the terpenoid synthases are responsible for generating the myriad different terpenoids produced by plants. These secondary transformations most commonly involve oxidation, reduction, isomerization, and conjugation reactions, which impart functional properties to the terpenoid molecules.

**Figure 7:**
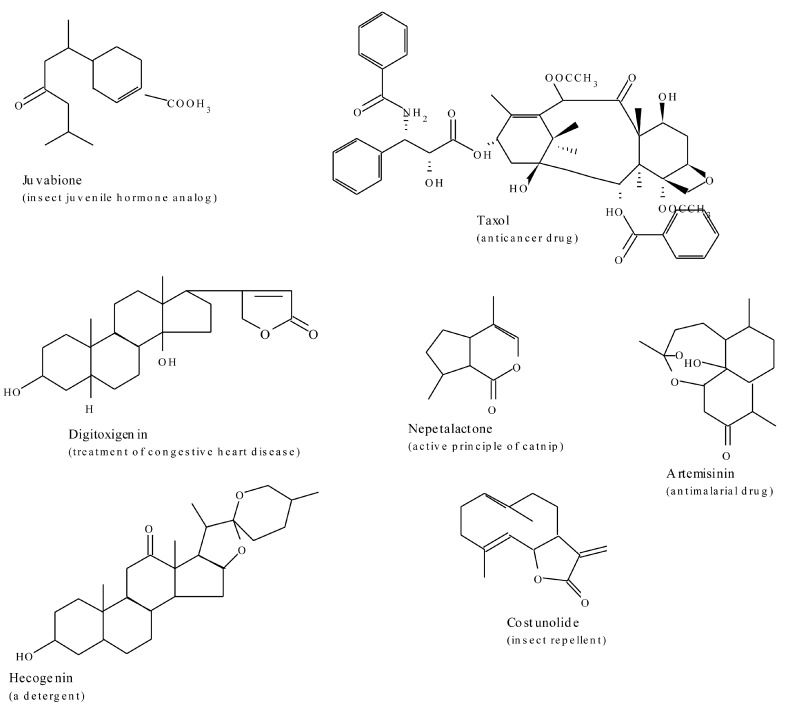
A few bioactive examples of the terpenoid group of plant derived secondary metabolites.

The chemical diversity of plant terpenoids is probably a reflection of their multiple biological activities in nature. In the producing plants, isoprenoids serve numerous biochemical functions that include: electron transport chains, as components of membranes (sterols), in subcellular targeting and regulation (prenylation of proteins), as photosynthetic pigments (carotenoids, side chain of chlorophyll), as hormones (gibberellins, brassinosteroids, abscisic acid, cytokinins), and as plant defence compounds as well as attractants for pollinators [98,102]. Artemisinin, an anti-malarial sesquiterpenoid isolated from *Artemisia annua*, and taxol, a high-value diterpenoid-derived anti-cancer drug from the bark of *Taxus brevifolia*, are some of the examples of pharmaceuticals derived from a family of terpenes. Artemisinin, in the form of combination therapies, is today the only effective treatment for multi-drug-resistant strains of the malaria parasite *Plasmodium falciparum*. Digitoxin, the glycone digitoxigenin extracted from foxglove (*Digitalis*), is used widely in carefully prescribed doses for treatment of congestive heart disease. Azadirachtin A is a powerful insect antifeedant terpenoid compound isolated from *Azadirachta indica*. A few examples of these are presented in Figure 7.

## 3. Metabolic Alterations 

After decades of elucidating secondary metabolic pathways, gene regulations, enzymes involved, and factors affecting various important metabolites, accumulated evidence has enabled, to a greater extent, the ability to model these systems and engineer plant metabolic pathways for enhanced metabolite production. A multitude of factors, the complex integrated regulatory mechanisms and coordinated networks of metabolic routes leading to the synthesis of specific metabolites, as well as the general plasticity and adaptability of the various biosynthetic pathways, shape the profiles and fluxes of plant secondary metabolite. Exploitation of the plant biosynthetic capacity presents numerous exciting opportunities but with equally complex challenges. Much of this rich chemical diversity arises from a limited pool of chemical scaffolds which are subsequently modified though specific chemical substitutions as catalysed by substrate and/or regio-specific enzymes. The enzyme-driven reactivity and regio- and stereo-chemistry during the multi-step conversion of substrates into precise products in the biocatalytic landscape of secondary metabolism is one of the lucrative key points of exploitation. The biomimetic exploitation of enzymes, particularly those that exhibit strict stereospecificity, is an interesting aspect in the production of improved pharmaceutical compounds. Equally intriguing is the biosynthesis of novel metabolites via protein engineering aimed at altering the substrate specificity of biosynthetic enzymes [28]. According to Bailey [103], application of recombinant DNA methods to restructure metabolic networks can improve production of metabolite and protein products by altering pathway distributions and rates. Recruitment of heterologous proteins enables extension of existing pathways to obtain new chemical products, alter posttranslational protein processing, and degrade recalcitrant wastes. Transgenic plants with altered enzyme activities have also become a powerful tool to study the metabolic control architecture of secondary metabolites.

Rational metabolic engineering, as defined by Bailey [104], involves the proposal of a defined genetic manipulation that is expected to provide a benefit via the perturbation of the known biochemical network, based on the knowledge of the metabolic system of interest. This definition emanates from the earlier definition coined from that of general metabolic engineering: “*the improvement of cellular activities by manipulation of enzymatic, transport, and regulatory functions of the cell with the use of recombinant DNA technology*” [103]. Broad and encompassing in their context as these definitions may be, their application in the truest sense sometimes results in metabolic consequences that deviate from those expected upon the genetic changes introduced. Against this background, Sauer [105] argues that the functional behaviour of a network emerges through the nonlinear gene, protein, and metabolite interactions across multiple metabolic and regulatory layers. Intracellular reaction rates are thus the functional end points of these interactions in metabolic networks, hence are highly relevant for systems biology. Beyond methods to quantify component concentrations, systems biology thus requires experimental methods for elucidating component interactions and quantitative monitoring of integrated network responses that result from the highly nonlinear interaction of the various components across functional levels [105,106,107,108]. The fact that secondary metabolite biosynthesis and accumulation remain under the influence of the environment further adds to the multiple dimensions of the metabolic manipulation level points for enhanced production. Following this direct logic, varied levels of metabolic perturbation through manipulation of environmental factors, either singularly or in combination, have been reported to trigger positive abrupt activation of qualitative and quantitative changes in plant secondary metabolite accumulation [2]. Other approaches entail diverting the carbon flux into a competitive pathway or an increase in the catabolism of the target compound. The biosynthesis of certain metabolites are under strict developmental regulation in plants, a characteristic attribute due to which exploitation of cultured cells for the production of certain valuable metabolites has been severely restricted.

Taken collectively, genomic and biochemical approaches and an appreciation of molecular evolution and environmental influence, as well as structural enzymology, holds great promise for altering the complex plant secondary metabolic pathways towards synthesis and accumulation of desired bioactive compounds. Notwithstanding the notable success of these efforts, complex cellular responses to metabolic perturbations often complicate predictive output and hence significant failures have equally been reported, perhaps more than the successes themselves. However, the regulatory architectures of these pathways, and the ways in which they are integrated into broader metabolic networks, are less well understood, often making it hard to predict the results of overexpressing a single gene or multiple genes within a particular pathway [109].

### 3.1. Metabolic Pathway Manipulations

Several attempts to dissect secondary metabolism for the purpose of improving bioactive metabolites using classical genetics have yielded, to some extent and in some species, positive results. Owing to the multitude of approaches employed in this regard, it would be impossible for this review to exhaustively discuss all the possible manipulation techniques applied to enhance production of fine chemicals. In this review we give an insight into some of these aspects and highlight a few examples in each case.

Understanding the basic network of metabolic intermediates and enzymes forms the fundamental basis in unravelling these attributes. Beyond this level, knowledge of the spatial and temporal regulatory architectures of secondary metabolic pathways, and the ways in which they are integrated into broader metabolic networks is key. It forms the focal point in the exploitation of TFs for predictive plant metabolic engineering. Transcription factors, a diverse group of proteins that recognise specific DNA sequences in the promoters of the genes, negotiate the regulation of gene expression at the level of transcription. TFs mediate the assembly of the basal transcription machinery resulting in the activation of RNA polymerase II and mRNA synthesis. The control of specific sets of genes within the metabolic network is accomplished by the combinatorial interaction among TFs, between TFs and non-DNA-binding proteins, and between TFs and *cis*-regulatory elements in an organised hierarchical gene regulatory networks TF [110,111]. Owing to their regulatory role in secondary metabolism, TFs add to the varied existing techniques, an efficient new molecular tool for plant metabolic engineering to increase the production of valuable compounds. Parallel to this, artificial TFs are also gaining momentum as valuable tools for plant metabolic engineering. The use of specific transcription factors would avoid the time-consuming step of acquiring knowledge about all enzymatic steps of the often poorly characterised biosynthetic pathways. Although manipulating the expression of specific transcription factors can modulate pathway flux, the complex mechanisms of transcriptional regulation can be an obstacle to obtaining the desired amount and/or balance of metabolites.

The traditional approach to engineering plant metabolic pathways has often been to target single slow or regulated steps that may limit pathway flux. Although this approach can be successful, identifying rate-limiting steps is often difficult. Beyond simple genetic metabolic manipulations that lend success to the absence of non-obvious limitations, well-articulated metabolic studies would enable us to devise new, non-obvious metabolic engineering strategies. Metabolic flux data rarely reveal a direct engineering target, primarily because fluxes that result from multiple component interactions and genetic manipulations must be made at the component level through, for example, overexpression of a gene [110]. Methods have also been developed for expressing complex multigene pathways for the biosynthesis of plant secondary metabolites in microorganisms. Some notable examples include, the successful expression of artificial three-gene clusters in *Escherichia coli* to make the plant flavanones pinocembrin and naringenin from phenylalanine and tyrosine, respectively [112]. Yeast has been engineered with two plant genes to produce the antimicrobial and protective stilbene resveratrol from fed 4-coumaric acid [113]. Martin *et al.* [114] reported that the expression of a synthetic amorpha-4,11-diene synthase gene and the mevalonate isoprenoid pathway from *Saccharomyces cerevisiae* in *Escherichia coli* led to concentrations of amorphadiene, the sesquiterpene olefin precursor to artemisinin, reached 24 μg caryophyllene equivalent/mL. Because isopentenyl and dimethylallyl pyrophosphates are the universal precursors to all isoprenoids, the strains developed in this study can serve as platform hosts for the production of any terpenoid compound for which a terpene synthase gene is available.

One of the powerful tools in phenotype-driven genetics that has been employed in plant metabolic engineering is gain-of-function mutagenesis with a strong constitutive promoter that is carried on an insertion element such as *Agrobacterium tumefaciens* T-DNA. In *Catharanthus roseus* cells, for example, T-DNA activation tagging of the octadecanoid-derivative-responsive ORCA3 transcription factors conferred resistance to a toxic derivative of the metabolic intermediate tryptamine in the terpenoid indole alkaloid pathway [30] TIA biosynthesis enzyme tryptophan decarboxylase [25]. Overexpression of ORCA3 upregulated several other genes, including genes in precursor pathways, in terpenoid indole alkaloid biosynthesis besides that encoding tryptophan decarboxylase which detoxifies tryptamine. Reverse genetic approaches have been used to change the nicotine content in *Nicotiana attenuata*. Virus-induced gene silencing of *PMT* genes by constructs with inverted repeat expressed from the CaMV 35S promoter led to reduced transcript levels in roots and reduced nicotine contents in leaves, whereas in contrast to wild-type plants, the silenced plants contained anatabine, which is presumably formed because of the accumulation of nicotinic acid, which is normally coupled to a downstream product of *PMT* to form nicotine [115]. A similar observation of elevated levels of anatabine at the expense of nicotine was made in *Nicotiana tabacum* plants expressing antisense PMT [116].

When a terminal enzyme in morphine biosynthesis, codeinone reductase, was knocked out by RNAi in *Papaver somniferum* using a chimeric inverted repeat that targets all seven members of the gene family, a significant reduction in the morphine and codeine levels of the transgenic poppy latex was recorded concurrently with a drastic switch in the alkaloid pattern, with the transgenic latex accumulating rare alkaloids [117]. These included the upstream precursor reticuline, which is located seven enzymatic steps before the codeinone reductase-mediated reaction in the biosynthesis pathway, and several methylated derivatives of reticuline. The incorporation of cDNA encoding hyoscyamine 6β-hydroxylase from *Hyoscyamus niger* to low-scopolamine (an anticholinergic agent) producing *Atropa belladonna* plants through the introduction of a constitutively expressed H6H transgene resulted in an increase in scopolamine accumulation [118]. Similarly, a shift in the accumulation of hyoscyamine in favour of scopolamine occurred when the H6H transgene was introduced into *Atropa beatica*, suggesting that the enzyme is a rate-limiting step in scopolamine biosynthesis. This represents one of the first successful examples of engineering a medicinal plant to produce a valuable end product. In *Brassica napus*, transformation with the cDNA encoding the *C. roseus* tryptophan decarboxylase involved in the biosynthesis of monoterpenoid indole alkaloids, has led to the redirection of tryptophan pools away from indole glucosinolate biosynthesis and into tryptamine [119], making the seeds more suitable for use as animal feed.

Transcriptional factors have also been used to drive flux through a pathway and the expression of an enzyme used to divert pathway intermediates to the desired final product. Examples in this application are illustrated by the production of isoflavones in tobacco, Arabidopsis, and maize, which normally do not accumulate these phytochemocals, by the coexpression of the flavonoid regulators with isoflavone synthase [120]. A similar approach was successfully employed to increase isoflavones in soybean, by combining the synthetic maize C1 and R TFs and the silencing of the endogenous *F3H* [121,122]. The potential benefit of using transcription factors to modify flux through a metabolic pathway is also highlighted by following two research findings that aimed to increase the concentration of health-beneficial flavonoids in tomato. The constitutively overexpressing of *CHI*, which encodes chalcone isomerase, an early flavonoid pathway enzyme that is expressed at low levels in tomato fruit, resulted in increased flux through the flavonoid pathway in the fruit, although, rather unexpectedly, fruit flavonols rather than anthocyanins increased [123]. Similarly, Bovy *et al* [124] overexpressed the maize anthocyanin regulators *Leaf colour* (*Lc*) and *C1* in a fruit-specific manner and observed the same accumulations pattern of metabolites. Collectively, these results underline that, in contrast to a pathway gene, a pathway activator can induce the accumulation of metabolites in a tissue in which most of the relevant enzymatic activities are normally insufficient.

When the microbial *ubiC* gene encoding chorismate pyruvate lyase was introduced into *Lithospermum erythrorhizon* hairy root cultures, which normally produce the naphtoquinone shikonin via the phenylalanine pathway, the chorismate-derived 4HB contributed to 20% of the overall 4HB production in the hairy roots and the levels of menisdaurin, a nitrile glucoside, were increased fivefold [125]. This example demonstrates that increasing the level of an intermediate may lead to the production of unexpected products. By the overexpression of the gene encoding H6H in *Hyoscyamus muticus* hairy root cultures, a 100-fold increase of scopolamine levels was reached compared with controls that produced hyoscyamine as the major alkaloid [126]. Not only were large amounts of scopolamine produced but also high levels of hyoscyamine accumulated in the hairy roots. Overexpression of the *Coptis japonica SMT* gene in a plant cell culture of *Eschscholzia californica*, a plant lacking this enzyme, resulted in the production of columbamine, which is normally not found in this species [127]. One fascinating aspect proved by this observation is that fluxes at a branch point can be changed by metabolic engineering. Opening up a new pathway at the intermediate scoulerine apparently channelled the flux away from the sanguinarine branch, resulting in considerably lower levels of this alkaloid. An interesting approach for the production of new compounds in a plant might be to introduce enzymes with a different substrate specificity (combinatorial biochemistry) [128] as outlined by these findings. These examples show that engineering a single functional gene has considerable value for metabolic engineering but also has some limitations. Although a substantial increase in productivity is feasible when a rate-limiting enzyme is targeted, single rate-limiting steps may not exist in most biosynthetic pathways. In most pathways, overexpression of one enzyme renders subsequent reactions more limiting. Strategies must, therefore, include modification of the multiple steps by overexpression of multiple biosynthetic genes, manipulation of regulatory genes that control the expression of multiple pathway enzyme genes, or both.

From the few empirical examples discussed herein, it is clear that metabolic engineering technology is feasible for secondary metabolism. However, all approaches to metabolic engineering require a thorough knowledge of the biosynthetic pathways involved. This is in fact one of the major limitations, as most pathways are not fully understood, with most of them only known at the level of intermediates. Thorough mapping of biosynthetic pathways thus becomes a prerequisite for any successful metabolic engineering programme. The enormous number of enzyme-catalysed reactions in secondary metabolism provides unprecedented opportunities for the selection of suitable enzymes for use in metabolic engineering. Although the potential of metabolically engineering plant-derived secondary metabolites is high, there have been few successes in modifying pathways to produce important beneficiary compounds. The impeding question, however, is how much of their metabolic and regulatory architecture do we know? Functional genomics approaches are perhaps some of the powerful tools that can be exploited for accelerating comprehensive investigations of theses biological systems. A great variety of plant-derived pharmaceutical compounds stand to benefit from yield improvement produced by genetic engineering if this limiting knowledge gap is filled.

Although plant cell and/or organ cultures are often used and economically feasible for production of certain compounds, optimisation of the culture environment for enhanced production is among the challenges to this innovation. Shikonin, taxol, and paclitaxel, for example, are perhaps the few compounds of plant cells so far produced on a commercial scale. Other products that came close are ginseng roots, rosmarinic acid, sanguinarine, and certain polysaccharide mixtures from cell cultures. Secondary metabolism is, by definition, a form of differentiation. The first and logical approach to improved production would thus be the culture of differentiated cells. A major limitation of differentiated organ cultures is their difficulty to cultivate on a large scale. As a result, their application is probably restricted to serve as models for studies of the regulation of the biosynthesis. Overcoming this hurdle could possibly unlock more potential for commercial secondary metabolites but requires further studies on the regulation of the differentiation process. The intracellular localisation of target products could also be another important target for metabolic engineering.

Empirical data show that there is a great potential for a broad range of applications, ranging from improving the production of certain secondary metabolites to the introduction of new pathways in plants. For further developing the full potential of metabolic engineering it is thus necessary to increase our knowledge about plant secondary metabolism, at the level of the intermediates, the enzymes and the genes. Understanding the physiology of the pathway is equally essential, as transport, pH, and cellular and subcellular compartmentation also play an important role [128]. Genomic sequencing of the target plant species using proteomics and metabolomics as tools for linking the genes with the secondary metabolite pathways would be a useful approach. 

### 3.2. Other Manipulated Factors

Manipulation of environmental plant stress factors such as light, mineral nutrition, water, temperature, salt stress, CO_2_, *etc.*, in mircoculture and/or related cultivation setups has previously been employed to tilt metabolism towards desired outputs, with varying degrees of success [129,130,131,132]. Eilert *et al.* [133] reported an accumulation of tryptamine and indole alkaloids, such as catharanthine, in *C. roseus* cell cultures following treatment with a fungal elicitor, and rapid induction of *TDC*, *STR*, and *SGD* gene expression has been reported [134], a phenomenon that suggested that the elicitor-mediated signal transduction pathway consists of relatively few steps that activate pre-existing transcription factors. Fungal elicitors also induced jasmonic acid (JA) biosynthesis in cell cultures of the same species, with jasmonate precursor α-linolenic acid or methyl jasmonate itself inducing *TD*C and *STR* gene expression when added exogenously [135].

As earlier mentioned herein, this review is not intended to exhaustively discuss all the possible metabolic alterations to improve on secondary metabolite biosynthesis. Against this backdrop, we further emphasise the multiplicity of the metabolic alteration approaches that can be employed in secondary metabolism as paralleled by the overly complex network of metabolic pathways leading to the biosynthesis of these compounds. In addition to the genetic, enzymatic, and transcriptome metabolic alteration techniques, numerous other approaches can be exploited that range from the use of elicitors (abiotic and biotic) in cell cultures to the manipulation of environmental factors including plant growth regulators at cellular, organ, and whole plant level. Table 2 presents a few examples of elicitor and environmental factors that have been manipulated to enhance production of bioactive secondary metabolites.

**Table 2:** Examples of factors that can be exploited to increase secondary metabolites in plants.

Metabolite	Plant Species	Factor Manipulated	Ref.
Morphine, codeine	*Papaver somniferum*	*Vertcillium dahliae*	[136]
Indole alkaloids	*Catharanthus roseus*	Fungal elicitor	[133,137]
Indole alkaloids	*Catharanthus roseus*	Diethyl amino ethyl dichloro phenyl ether	[138]
*N*-acetyl-tryptamine	*Catharanthus roseus*	*Pythium aphanidermatum*	[139]
Ajmalicine	*Catharanthus roseus*	*Trichoderma viride*	[140]
Catharanthine	*Catharanthus roseus*	Vanadium sulphate	[141]
Camalexin, indole glucosinolates	*Arabidopsis thaliana*	*Erwinia carotovora*	[142]
Camalexin	*Arabidopsis thaliana*	Oxidative stress, amino acid starvation	[143]
Diterpenoid tanshinones	*Salvia miltiorrhiza*	Yeast elicitor	[144]
Rutacridone epoxide	*Ruta graveolens*	Chitosan	[145]
Silymarin	*Silybum marianum*	Yeast extract, Methyl jasmonate	[146]
Rosmarinic acid	*Coleus blumei*		[147]
Saponins	*Panax ginseng*	Low-energy ultrasound	[148]
Diosgenin	*Dioscorea deltoida*	*Rhizopus arrhizus*	[149]
Hyoscyamine, scopolamine	*Hyoscyamus niger*	Fungal elicitor,	[150]
Hyoscyamine, scopolamine	*Hyoscyamus* *muticus*	Fungal elicitor, Methyl jasmonate	[150]
Salidroside	*Rhodiola sachalinensis*	*Aspergillus niger*, *Coriolus versicolor*, *Ganoderma lucidum*	[151]
Sanguinarine	*Papaver bracteatum*	Dendryphion	[152]
Taxol	Taxus chinensis	Fungal elicitor	[153]
Tropane alkaloids	*Brugmansia suaveolens*	*Spodoptera frugiperda*, Methyl jasmonate	[154]
Scopoletin	*Ammi majus*	*Enterobacter sakazaki*	[155]
Tanshinone	*Salvia miltiorrhiza*	Hyperosmotic stress, yeast elicitor	[156]
Acridone expoxide	*Ruta gravelones*	Fungal poly saccharide	[145]
Colchicine	*Valeriana wallichii*	Valepotriates	[157]
Sesquiterpenoids	*Datura stramonium*	Metal ions	[158]
Capsidiol, debneyol, scopoletin, nicotine	*Nicotiana tabacum*	Phytopthora cryptogea, Yeast extract, Cryptogein Cellulase, Methyl jasmonate	[159,160,161]
Raucaffrincine	*Rauwolfia canescens*	Yeast elicitor, Methyl jasmonate	[162,163]
Kinobeon A	*Carthamus tinctorius*	Blue green algae	[164]
Isoflavonoids	*Lotus corniculatus*	Glutathione	[165]
Digoxin, Purpureaglycoside A	*Digitalis lanata*	Temperature	[166]
Ubiquinone	*Nicotiana tabacum*	Temperature	[167]
Crude alkaloids	*Catharanthus roseus*	Temperature	[168]
Anthocyanin	*Daucas carota*	Light	[169]
Sesquiterpenes	*Marticaria chamomilla*	Light	[170]
Monoterpenes	*Citrus limo*	Light	[171]
Catechin, epicatechin,	*Malus × domestica* Borkh	Chilling	[172]
Flavonoids, tannins	*Cyrtanthus contractus*, *C. guthrieae*	Sodium chloride	[173]
Digitoxin	*Digitalis purpurea*	Phosphate	[174]
Betacyanin	*Chenopodium rubrum*	Phosphate	[174,175]
Betacyanin	*Phytolacca americana*	Phosphate	[38,176]

## 4. Conclusions

Plant secondary metabolites represent enormous chemical diversity with largely unexplored pharmacological activities. Our extensive knowledge on the chemistry and pharmacology of some secondary metabolites has led to their use in a range of medical applications. The wide chemical diversity of secondary metabolites throughout the plant kingdom, therefore, represents an extremely rich biogenic resource for the discovery of novel and innovative drugs. The fact that no general conclusions can be drawn about the chance that a certain approach will be successful or the outcome predicted with certainty is what makes plant secondary metabolic alterations for novel and innovative bioactive compounds a lucrative aspect and offers limitless opportunities in the metabolism itself. This, therefore, positions plants, through their plastic, complex and diverse secondary metabolism, as natural chemical factories able to carry out combinatorial chemistry that mankind exploits to his benefit. In several cases, overexpression results in the production of unexpected products, demonstrating the complexity of the metabolic networks and our knowledge gap of these networks and their regulation. Owing to their ability to control both multiple pathway steps and cellular processes that are necessary for metabolite accumulation, transcription factors, among other techniques, offer much promise for the manipulation of metabolic pathways. This is particularly true of complex pathways whose component enzymes are poorly characterised. Notwithstanding the significant contributions of other factors and metabolic alteration approaches to metabolite production, the pleiotropic action of central transcription factors on a wide array of genes involved in metabolic differentiation of plant cells, enable the development of new strategies to engineer complex metabolic pathways and thus holds great promise for increasing the level of pharmaceutically active compounds.
